# The Perturbation of Pulmonary Surfactant by Bacterial Lipopolysaccharide and Its Reversal by Polymyxin B: Function and Structure

**DOI:** 10.3390/ijms19071964

**Published:** 2018-07-05

**Authors:** Maros Kolomaznik, Gilda Liskayova, Nina Kanjakova, Lukas Hubcik, Daniela Uhrikova, Andrea Calkovska

**Affiliations:** 1 Martin Biomedical Center and Department of Physiology, Jessenius Faculty of Medicine in Martin, Comenius University in Bratislava, 036 01 Martin, Slovakia; maros.kolomaznik@uniba.sk (M.K.); andrea.calkovska@jfmed.uniba.sk (A.C.); 2 Department of Physical Chemistry of Drugs, Faculty of Pharmacy, Comenius University in Bratislava, 832 32 Bratislava, Slovakia; liskayova@fpharm.uniba.sk (G.L.); kanjakova2@uniba.sk (N.K.); hubcik@fpharm.uniba.sk (L.H.)

**Keywords:** pulmonary surfactant, surface activity, inhibition, lipopolysaccharide, polymyxin B, SAXS

## Abstract

After inhalation, lipopolysaccharide (LPS) molecules interfere with a pulmonary surfactant, a unique mixture of phospholipids (PLs) and specific proteins that decreases surface tension at the air–liquid interphase. We evaluated the behaviour of a clinically used modified porcine pulmonary surfactant (PSUR) in the presence of LPS in a dynamic system mimicking the respiratory cycle. Polymyxin B (PxB), a cyclic amphipathic antibiotic, is able to bind to LPS and to PSUR membranes. We investigated the effect of PxB on the surface properties of the PSUR/LPS system. Particular attention was paid to mechanisms underlying the structural changes in surface-reducing features. The function and structure of the porcine surfactant mixed with LPS and PxB were tested with a pulsating bubble surfactometer, optical microscopy, and small- and wide-angle X-ray scattering (SAXS/WAXS). Only 1% LPS (*w*/*w* to surfactant PLs) prevented the PSUR from reaching the necessary low surface tension during area compression. LPS bound to the lipid bilayer of PSUR and disturbed its lamellar structure by swelling. The structural changes were attributed to the surface charge unbalance of the lipid bilayers due to LPS insertion. PxB acts as an inhibitor of structural disarrangement induced by LPS and restores original lamellar packing, as detected by polarised light microscopy and SAXS.

## 1. Introduction

Bacterial lipopolysaccharide (LPS), also termed endotoxin, is the major outer surface membrane component present in almost all Gram-negative bacteria. *In vivo*, it stimulates innate immunity and initiates a cascade of biochemical and cellular events leading to local inflammation and systemic toxicity of pathogens [1]. Upon inhalation, LPS interferes with a pulmonary surfactant—a unique mixture of phospholipids (PLs) and surfactant-specific proteins (SPs)-A, -B, -C, and -D—which decreases surface tension at the alveolar air–liquid interphase [2,3]. Along with SP-A and -D, which are members of the collectins family, hydrophobic surfactant protein SP-C also binds to the lipid A region of LPS [4,5]. LPS may also incorporate into lipid membranes of endogenous and, under certain clinical conditions, exogenous surfactants [6], resulting in surfactant dysfunction [7,8].

Polymyxin B (PxB) is an antimicrobial peptide primarily used in clinical practice to treat infections by resistant Gram-negative bacteria. Its structure (Supplementary Materials, Figure S1) includes a cyclic amphipathic decapeptide with five positively charged side chains and an acyl chain at the N-terminus [9]. In experimental settings, PxB mimics functional properties of surfactant protein B by cross-linking the lipid membranes [10]. We have previously shown that the addition of PxB improves the surface properties of exogenous surfactants and increases the resistance to inactivation with plasma albumin that enters the airspaces and interferes with pulmonary surfactants in the case of alveolar–capillary membrane damage [11]. The combination of surfactant/PxB or PxE mixtures was also tested in other models of surfactant inhibition, as in meconium aspiration syndrome or neonatal pneumonia [12,13].

The effect of LPS on the biophysical properties of surfactants has mainly been studied with the monolayers model, using relatively simple lipidic mixtures (e.g., [14,15]). To our knowledge, it has not been studied for the modified surfactant Curosurf, which is used to treat neonatal respiratory distress syndrome (RDS) of prematurity. This surfactant preparation is obtained from minced porcine lungs by organic extraction and is purified by liquid-gel chromatography. It is composed of about 99% (*w*/*w*) polar lipids, mainly PLs, and 1–2% (*w*/*w*) SP-B and -C. Zwitterionic phosphatidylcholines are the predominant PLs (up to ~75% *w*/*w*), and saturated dipalmitoylphosphatidylcholine (DPPC) constitutes up to 50% of this fraction [16,17]. Structurally, the modified porcine surfactant is a mixture of various vesicles, from unilamellar to multilamellar [17].

Recent research work on the structure of pulmonary surfactant layers has been devoted to analysis of the lateral in-plane organisation of lipids and proteins in membranes and interfacial films [18]. It was shown that interfacial films formed from native-like concentrated surfactant suspensions at relative rapid compression rates reach the lowest tension with less compression. A satisfactorily high concentration of the surfactant in the hypophase has been shown to produce interfacial films formed by several PL layers tightly associated with the interface [19]. The existence of a multilayer stack associated with the interface has been visualised by electron microscopy of lung tissue [19,20]. Interfacial properties of pulmonary surfactant films formed by lipid monolayers have been evaluated by different experimental techniques, including X-ray and neutron reflectometry (see [2] for a review). However, much less attention has been devoted to the study of lamellar structures associated with the air–water interface.

The aim of our study is to investigate the behaviour of the modified porcine pulmonary surfactant (PSUR) in the presence of LPS in a dynamic system mimicking the respiratory cycle. In addition, given that PxB is able to bind to LPS [21] and to PSUR membranes [10], we investigated whether the surface properties of the surfactant in the presence of LPS could be influenced by the addition of PxB. Particular attention was paid to mechanisms underlying the structural changes in lamellar structures that are recognised as “nanosilos”, associated with surface-reducing features. Optical microscopy and small- and wide-angle X-ray scattering (SAXS/WAXS) were employed in the structural study.

## 2. Results

### 2.1. Surface Activity

#### 2.1.1. Effect of Lipopolysaccharide

**Figure 1:**
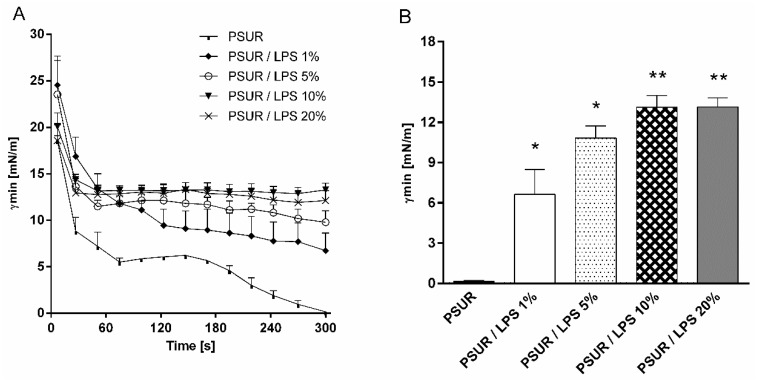
Minimum surface tension (*γmin*) of modified porcine surfactant (PSUR) at phospholipid concentration of 1.75 mg/mL without or with lipopolysaccharide (LPS) at 1%, 5%, 10%, and 20% (*w*/*w*) during the whole period of pulsation (**A**), and at fifth minute of pulsation (**B**) in pulsating bubble surfactometer. Values are given as means ± SEM. PSUR vs. PSUR/LPS: * *p* < 0.05 and ** *p* < 0.01.

Figure 1A shows the dynamic changes in the minimum surface tension (*γmin*) of PSUR and PSUR/LPS mixtures during the whole period of bubble pulsation in a surfactometer. After 5 min of pulsation, *γmin* values of all PSUR/LPS mixtures were significantly larger in comparison to the surfactant without LPS (all had *p* < 0.05–0.01 vs. surfactant), suggesting inactivation of the surfactant (Figure 1B). An inhibitory effect was already present at the LPS concentration of 1%. Increasing the LPS concentration up to 20% had no further influence on *γmin* (Figure 1B).

#### 2.1.2. Effect of Polymyxin B

**Figure 2:**
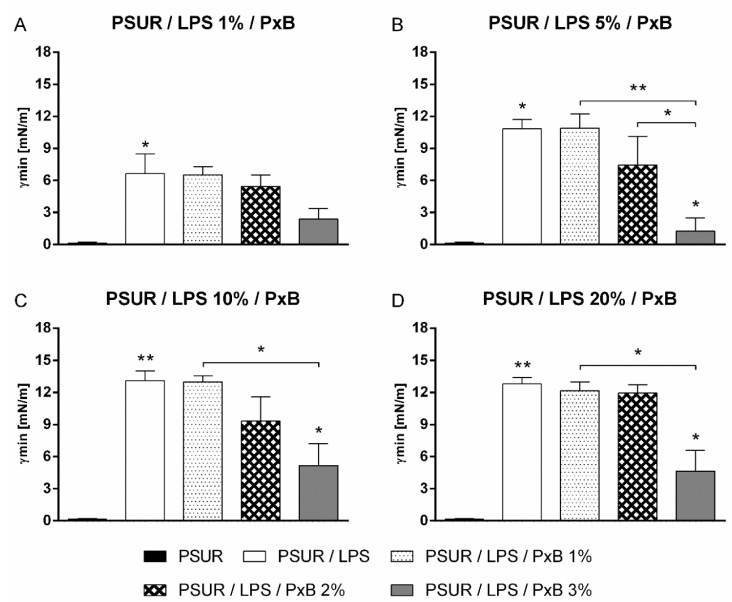
Minimum surface tension (*γmin*) of modified porcine surfactant (PSUR) at phospholipid concentration of 1.75 mg/mL with lipopolysaccharide (LPS) at 1% (*w*/*w*) and polymyxin B (PxB) at 1%, 2%, and 3% (**A**); LPS at 5% and PxB at 1%, 2%, and 3% (**B**); LPS at 10% and PxB at 1%, 2%, and 3% (**C**); and LPS at 20% and PxB at 1%, 2%, and 3% (**D**) after 5 min of pulsation. Values are given as means ± SEM. PSUR vs PSUR/LPS; PSUR/LPS vs PSUR/LPS/PxB: * *p* < 0.05 and ** *p* < 0.01.

The addition of PxB at concentrations of 1% and 2% did not prevent inactivation of the porcine surfactant by LPS. This even applied at the lowest LPS concentration of 1%, which means that there was no statistically significant reduction of *γmin* after 5 min of pulsation (*p* > 0.05 vs. PSUR/LPS) (Figure 2A). The *γmin* value of the PSUR/LPS mixture with 3% PxB was significantly reduced at LPS concentrations of 5%, 10%, and 20% (*p* < 0.05 vs. PSUR/LPS) (Figure 2B–D). The addition of 3% PxB to the PSUR/LPS mixture reduced *γmin* to values of less than 5 mN/m at 1% LPS, indicating a biophysically active surfactant. However, this change was not statistically significant (Figure 2A). There was also a significant decrease in the *γmin* values of the PSUR/LPS mixture with 3% PxB for all other concentrations of LPS (5–20%) (Figure 2B–D) (*p* < 0.01 vs. PSUR/5% LPS/1% PxB; *p* < 0.05 vs. PSUR/10% and 20% LPS/1% PxB) as well as for PSUR/5% LPS/1% PxB versus PSUR/5% LPS/2% PxB (*p* < 0.05; Figure 2B).

We did not observe any change in the surface tension when PxB was added to the PSUR itself. For all samples, the *γmin* values of the PSUR/PxB mixture were <5 mN/m.

Dynamic changes in the *γmin* values of the PSUR, PSUR/LPS, and PSUR/LPS/PxB diverse mixtures during the whole period of pulsation are shown in the Supplementary Materials, Figure S2A–D. During the pulsation, the maximum surface tension was relatively stable; we found that *γmin* decreased with increasing PxB concentration (Supplementary Materials, Figure S3A–D).

### 2.2. Structural Study

#### 2.2.1. Modified Porcine Surfactant

**Figure 3:**
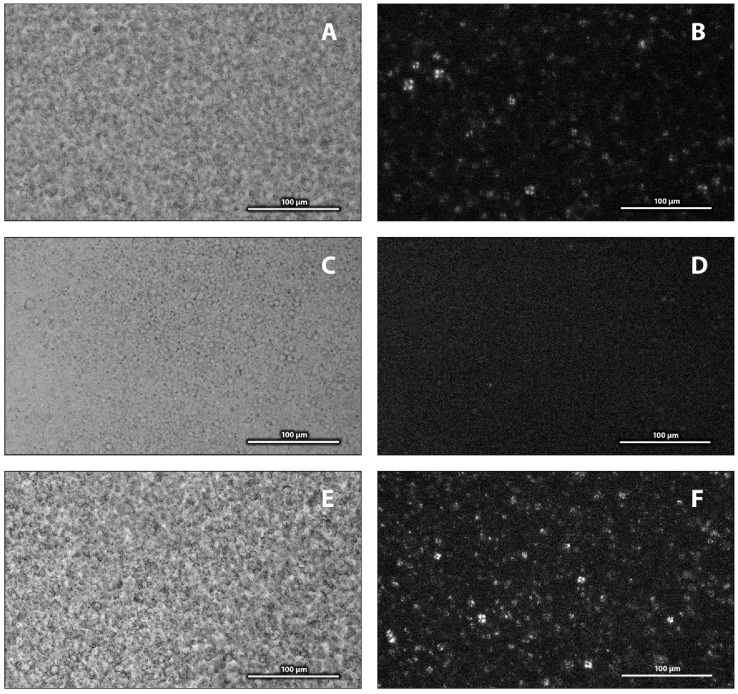
Photographic images recorded in normal and polarised light for aqueous dispersions of modified porcine surfactant (PSUR) (**A**,**B**), PSUR and 10% lipopolysaccharide (LPS) incubated for 2 h at 37 °C (**C**,**D**), and PSUR/10% LPS and 3% PxB (**E**,**F**).

Freeze-fracture electron micrographs of the PSUR (Curosurf) showed a mixture of various vesicles, from unilamellar through oligolamellar up to multilamellar (“onion structure”) [17]. Polarised light microscopy identified liquid-crystalline phases by following the textures. Figure 3A shows a microscope image of an aqueous dispersion of the surfactant. It corresponds to a non-homogeneous mixture. The same sample observed with polarised light (Figure 3B) depicted “Maltese crosses”, typical of multilamellar structures, similarly to the results of Larsson et al. [22]. Isotropic solutions, such as those of micelles or unilamellar vesicles, and the stiff transparent isotropic cubic phases are non-birefringent; thus only a black background with no well-characterised texture was observed.

**Figure 4:**
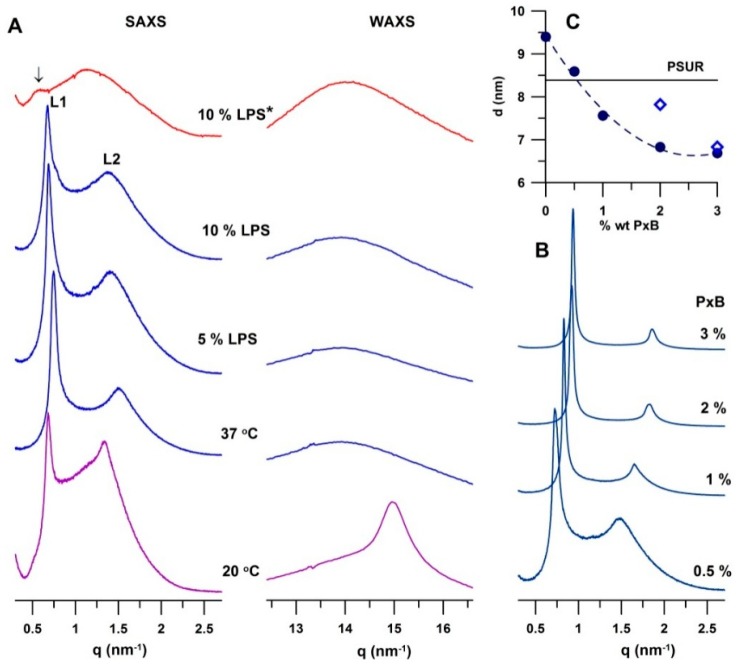
(**A**) Small- and wide-angle X-ray scattering (SAXS/WAXS) patterns of the modified pulmonary surfactant (PSUR) at 20 and 37 °C, and PSUR incubated with either 5% or 10% LPS for 30 min and 2 h (pattern at the top, marked with asterisk). An arrow indicates the first order peak of a lamellar phase with *d* ~ 12.6 nm; (**B**) SAXS patterns of PSUR/10% LPS/PxB, for which samples were incubated for 30 min; (**C**) the dependence of the repeat distance of PSUR/LPS/PxB system as a function of PxB concentration. Empty symbols are related to *d* of PSUR/10% LPS/PxB after longer incubation (2 h). Solid line represents the repeat distance of PSUR without any additives.

Figure 4A shows SAXS and WAXS patterns of the PSUR and PSUR incubated with LPS under different conditions. Two peaks *L*(*h*), where *h* = 1, 2 is the Miller index, were identified as reflections of a lamellar phase. At 20 °C, the surfactant formed a gel lamellar phase (*L_β_*). We determined the repeat distance as *d* = 9.21 ± 0.01 nm. A detailed inspection of the pattern revealed the presence of a broad peak of low intensity at *q* ~ 1.1 nm^−1^ that resulted in an increase in the baseline between the two peaks. Unilamellar and/or oligolamellar vesicles present in a dispersion can contribute to the SAXS pattern as a broad peak. WAXS gives information about PL acyl chains packing in the hydrophobic region of the bilayer. A symmetrical peak at *q* ~ 15 nm^−1^ is characteristic of the arrangement of lipid acyl chains perpendicular to the surface of the bilayer (gel *L_β_* phase) [23]. At a higher temperature, 37 °C, we measured the repeat distance as *d* = 8.35 ± 0.02 nm. A broad peak in the WAXS region is typical of the liquid-crystalline *L_α_* phase. Structural changes in the surfactant, induced by LPS and PxB, were followed at 37 °C, in the *L_α_* phase. WAXS patterns of all studied mixtures at 37 °C exhibited a broad peak in the range of *q* ~ 11–16 nm^−1^ with a maximum at *q* ~ 14 nm^−1^, characteristic of lipids with liquid-like acyl chains packed in a quasi-hexagonal lattice with a spacing of ~0.45 nm, in accord with observations of Larsson et al. [24].

#### 2.2.2. Effect of Lipopolysaccharide

LPS at both studied concentrations (5% and 10%) affected the multilamellar packing of the PSUR. Microscope images recorded after 30 min of incubation (at 37 °C) showed a more uniform texture including spherical objects. Polarised light revealed the presence of multilamellar structures, although their population was smaller (Supplementary Materials, Figure S4). Images of the same mixture recorded after a longer incubation (~2 h) are shown in Figure 3C,D. They indicate a further decrease in multilamellar structures, as seen particularly under polarised light, shown in Figure 3D. SAXS experiments revealed an increase in the repeat distance *d* of the PSUR induced by LPS. The effect depended on both the LPS content and incubation time. Figure 4A shows structural changes observed in the PSUR/LPS mixture in the *L_α_* phase. At both concentrations, 5 and 10 wt % of LPS, we detected an increase in the repeat distance *d*. It changed from 8.35 to 9.16 nm at 5% LPS and to 9.40 nm when the surfactant was incubated with 10% LPS for 30 min. A longer incubation (~2 h) generated a greater swelling up, which corresponded to a large unresolved SAXS peak of low intensity with a subtle shoulder at the low value of *q* ~ 0.5 nm^−1^ (marked by an arrow in Figure 4A, top). The pattern indicated swelling of a lamellar phase up to a periodicity of ~12–13 nm. It is worth mentioning that 5% LPS induced a similar effect (Supplementary Materials, Figure S5). WAXS patterns showed a liquid-like organisation of the acyl chains in the hydrophobic region of the bilayer in all studied mixtures.

#### 2.2.3. Effect of Polymyxin B

Figure 3E,F shows microscope images of PSUR/10% LPS and 3% PxB. The texture of the mixture was very similar to that observed for the pure surfactant (Figure 3A). The image, recorded with polarised light, showed a mixture rich in multilamellar structures (Figure 3F). The observed renewal of the lamellar packing called for a more detailed structural study using SAXS, as shown in Figure 4B,C.

Figure 4B displays the SAXS patterns of the PSUR incubated for 30 min with 10% LPS and consecutively with PxB. Even a small dose of the antibiotic reduced the repeat distance. Indeed, in the presence of 0.5% PxB, PSUR/LPS showed a lamellar phase with the repeat distance of *d* = 8.59 ± 0.01 nm (pattern at the bottom), which represents a reduction of *d* ~ 0.80 nm in comparison to its value prior to treatment. The repeat distance further decreased with the increasing content of PxB. Figure 4C summarises the observed changes in *d* of the PSUR/10% LPS mixture incubated for 30 min. The solid line in the plot indicates the repeat distance (*d* = 8.35 nm) of the lamellar phase of the PSUR prior to LPS application. PxB appeared as an effective modulator of lamellar arrangement; *d* decreased non-linearly to *d* ~ 6.83 nm, induced by 2% PxB. Further increasing the PxB content (to 3%) caused only a minor effect, the repeat distance being reduced to *d* = 6.69 ± 0.01 nm. As shown above, after a long incubation (2 h), LPS destroyed the lamellar packing of the PSUR, and the system swelled up to *d* ~ 12–13 nm (Figure 4A, top; Supplementary Materials, Figure S5). We recovered the regular lamellar packing, *d* = 7.82 ± 0.03 nm, when 2% PxB was added. The increase in PxB to 3% reduced the repeat distance to *d* = 6.83 ± 0.01 nm (empty symbols in Figure 4C). SAXS/WAXS patterns are shown in the Supplementary Materials, Figure S5.

## 3. Discussion

The exogenous surfactant isolated from porcine lungs (PSUR, trade name Curosurf) is effectively used in the treatment of RDS. We tested, *in vitro*, the effect of LPS and consecutive treatment with PxB on the function of the pulmonary surfactant. Our system mimicked alveolar expansion/compression during the respiratory cycle using a pulsating bubble surfactometer. Structural changes were examined by SAXS and WAXS. The study performed with the exogenous surfactant of animal source reflected the situation with a native lung surfactant more accurately than a model for membranes of synthetic lipids does. 

### 3.1. Effect of Lipopolysaccharide

The *in vitro* model of lung alveoli consisted of an oscillating air bubble acting in the liquid sample (PSUR). It mimicked inhalation (decompression) and exhalation (compression) of the surface area [24]. First, we evaluated the lowest concentration at which the PSUR was still active while remaining sensitive to inactivation by LPS, which occurred at a concentration of 1.75 mg of surfactant PLs/mL, which was within the range 1–5 mg of PLs/mL used in previous studies [25,26]. This value was close to the “critical concentration” of PLs (about 3 mg/mL) in foetal lung liquid at birth, needed for rapid absorption and optimal dynamic characteristics of the surfactant [27]. After 5 min of bubble pulsation in the surfactometer, both the minimum (*γmin*) and maximum (*γmax*) surface tensions of the PSUR/LPS mixtures were significantly higher than for the PSUR without LPS. We found that 1% LPS was sufficient to impair the function of the surfactant. Increasing concentrations of LPS, beyond 10%, did not lead to a further increase in *γmin*, meaning that, at a low surfactant concentration, a relatively low dose of LPS was necessary to impair surfactant activity. The PSUR/LPS ratio in our study was similar to the ratio used in the study of Brogden et al. [28], 64:1 (*w*/*w*), although it was much higher than with other surfactant inhibitors, such as plasma proteins [29] or meconium [12], because of other applied inhibitory mechanisms.

The complexing of LPS and the PSUR alters the properties of a surfactant and contributes to the pathophysiological changes observed in some types of pneumonia [7,28]. The exact mechanism by which LPS interferes with the surfactant layer at the alveolar surface has not been fully elucidated. It was shown that Re-LPS, the minimal LPS form required for bacterial growth, interacts with DPPC films, and as a result, DPPC monolayers become more fluid, and their surface tension properties are altered [30]. Similar effects were observed with more complex surfactant-like films made of various synthetic PLs. Cañadas et al. [15] evaluated the effect of the whole LPS molecule on the biophysical properties of lung surfactant-like films composed of either DPPC or DPPC/palmitoyloleoylphosphatidylglycerol (POPG)/palmitic acid (PA) (28:9:5.6, *w*/*w*/*w*). In their study, low amounts of smooth LPS fluidised DPPC monolayers and prevented them from achieving low surface tension during area compression, as occurred in our pulsating bubble study in the presence of LPS. 

Our SAXS/WAXS experiments revealed significant changes in the lamellar arrangement of the PSUR dispersion after incubation with LPS. PSUR (Curosurf) is composed of approximately 99% lipids, mainly PLs, and small amounts of hydrophobic surfactant proteins SP-B and -C. It consists of ~75 wt % phosphatidylcholines, half of which is DPPC [16]. DPPC is saturated phosphatidylcholine that forms a lamellar phase with a well-characterised temperature behaviour: a tilted gel phase (*L_β’_*) below 35 °C, a rippled gel phase (*P_β_*) below 42 °C, and a liquid-crystalline phase (*L_α_*) above 42 °C [31]. Typical SAXS/WAXS patterns of fully hydrated DPPC are shown in Figure S6 (Supplementary Material). The weight fraction of DPPC may vary depending on the surfactant’s origin, and its content affects the temperature behaviour. As such, temperatures of the phase transition (*T_m_*) from the gel to liquid-crystalline state are scattered between 33 and 38 °C. The presence of a significant amount of unsaturated PL species in the surfactant reduces the melting temperature (*T_m_*) to values lower than that of pure DPPC, making surfactant membranes fluid at physiological temperatures [18,22,32,33]. The WAXS patterns in Figure 4A show that the surfactant (PSUR) used in our experiments formed a gel phase *L_β_* at 20 °C and a fluid liquid-crystalline *L_α_* phase at 37 °C. All experiments were performed at 37 °C. The PSUR showed, at 37 °C, a liquid-crystalline lamellar phase (*L_α_*) with a repeat distance of *d* = 8.35 ± 0.01 nm. For comparison, DPPC itself, at 37 °C, is in a gel state and forms a rippled phase with *d* ~ 7.3 nm, which drops to *d* ~ 6.7–6.6 nm in the *L_α_* phase (above 42 °C) [34]. Generally, *d* values of zwitterionic phosphatidylcholines in the *L_α_* state do not exceed ~6.5–6.7 nm [35]; *d* is the sum *d* = *d_L_* + *d_w_*, where *d_L_* is the thickness of the lipid bilayer and *d_w_* is the thickness of the water layer between two neighbouring lipid bilayers. For zwitterionic phosphatidylcholines, the thickness *d_w_* ~ 1.8–2 nm is the result of a balance between repulsive interbilayer interactions (steric, hydration, and fluctuation) and attractive van der Waals forces [35]. The presence of any charges localised on the surface of the lipid bilayer (e.g., charged PLs) results in electrostatic repulsion between the two neighbouring bilayers, and the lamellar structure swells. Anionic PLs (phosphatidylglycerol and phosphatidylinositol) represent up to 8–15% of the weight of a pulmonary surfactant. In addition to lipids, proteins SP-B and -C contribute to the resulting surface charge of the bilayers, although their content does not exceed ~2 wt %. SP-B has 79 residues, with a net positive charge of +7; it is a member of the saposin family and is expected to adopt the characteristic saposin fold containing four or five α-helices connected by unstructured loops [36]. SP-C is a very hydrophobic protein; it has 35 residues and a net positive charge of +3 and forms an α-helix [37]. Both proteins are anchored in the hydrophobic core of the bilayer with their charged hydrophilic residua oriented towards the aqueous phase, thus contributing to the total surface charge of the membrane. Zeta potential measurements show the PSUR (Curosurf) as a dispersion with a negative total charge under physiological conditions [38]. The steric thickness of the DPPC lipid bilayer in the liquid-crystalline state is ~4.5 nm [35,39]. Larsson et al. [24] reported the lipid bilayer thickness of *d_L_* ~ 3.8 nm in a lamellar phase with a spacing of 8.8 nm for a bovine lung surfactant at 40 °C. This short analysis indicated that the thickness of the water layer, *d_w_*, could reach up to ~3.9–4.6 nm in the lamellar phase observed in our PSUR dispersion. Such a large water gap allows for higher freedom in the molecular and bilayer fluctuations, which induce disorder in the “long-range organisation” and reduce the intensities of higher-order reflections in the diffraction pattern. Weak binding between individual lamellae can distort the multilamellar arrangement, contributing also to the observed higher baseline between the two peaks of the SAXS patterns.

Both methods, microscopy and SAXS, showed that LPS disturbed the lamellar packing in the dispersion of the PSUR. Augusto et al. [4] reported a high binding affinity of LPS to the SP-C protein, particularly while immersed in a lipid environment. LPS intercalates into PL liposomes in the presence of divalent cations and/or after long-term incubation (up to 72 h) at 37 °C [40]. Our microscopic observations indicated a drop in the population of multilamellar structures after 2 h of incubation of the PSUR/LPS mixture at 37 °C (Figure 3D). Negatively charged lipids, such as POPG, suppress the diffusion of LPS in the bilayer composed of egg—PC and POPG [41]. LPSs show a tendency for the formation of non-lamellar phases. For example, cubic phases were detected in the presence of divalent cations [42,43,44]. Nevertheless, a cubic phase exists for LPS interaction with a DPPE/DPG mixture only at a very high content (non-physiological) of LPS [45]. Nomura et al. [41] observed local defects of the PC/PG membrane due to LPS binding and proposed a model for the cubic arrangement of the LPS/lipid mixture attached to the giant vesicle.

Figure 4A and Figure S5 show that the multilamellar structure of the PSUR swelled after a longer incubation with LPS. The observed broad, non-resolved peaks indicated weak correlations between lamellae. Such an SAXS pattern (Figure 4A, top) may result also from the superposition of two or more cubic phases or the “sponge phase”, made by a deformed three-dimensional structure. However, the image under polarised light showed the presence of a lamellar arrangement, which was less evident after a long incubation (Figure 3F and Figure S4). The cubic phases of lipid systems are known, with their high viscosity. Macroscopically, they form transparent gel-like particles that we did not observe in our samples. To conclude, our experimental results are in favour of a lamellar packing that markedly swells as a result of LPS intercalation into the lipid bilayer.

The observed changes in surface tension and structure were in accord with the concept of LPS being intercalated into PSUR bilayers. Bacterial endotoxin LPS molecules consist of a hydrophobic lipid (lipid A), a large hydrophilic polysaccharide chain called a core, and a long O-antigen that represents the outermost domain exposed to the water phase. The intercalation of the endotoxin’s robust molecule into the PSUR bilayer affects the lipid/water interface: the average area per lipid molecule increases as a result of the additive itself and because of the electrostatic repulsion between the negatively charged LPS and phosphate fragments of polar headgroups of PSUR PLs (phosphatidylcholines and phosphatidylglycerol). In a first approximation, the reduction of the surface tension (*γ*) at the air/water interface induced by amphiphilic surfactant can be expressed as

$$\gamma =\gamma_{0}-\pi \left(A_{L}\right),$$

where *γ*_0_ is the surface tension of the pure interface (~70 mN/m at 37 °C for air/water), *A_L_* is the area per lipid molecule, and *π(A_L_)* is the surface pressure arising from the interactions between molecules [46]. The surface tension at the interface depends on the molecular surface density, which is inversely proportional to the area per lipid. The higher the surface density (and the lower the area per lipid), the lower the surface tension at the interface. Thus, an increase in the average *A_L_* of LPS/PSUR reduces the molecular surface density, which in turn increases the surface tension *γ*.

### 3.2. Effect of Polymyxin B

In this study, we used PxB, an antibiotic isolated from *Bacillus polymyxa*, to reverse LPS-induced PSUR inactivation. PxB is mainly used for the topical treatment of Gram-negative infections, and one of the reasons for its use here was its ability to neutralise the harmful effects of the endotoxin [47]. The mechanism of action of polymyxins, and particularly of PxB, is known [48]. The initial target of its antimicrobial activity is anionic LPS in the outer membrane of Gram-negative bacteria. The high affinity for LPS binding can be attributed to the polycationic character of PxB. Charge, together with the amphipathic character of the molecule, allow the intercalation of PxB into the lipid bilayer and its ability to form pores, which leads to bacteriolysis in lipid membranes [21,49].

Another motivation to use PxB was its known ability to prevent surfactant inactivation by inhibitors other than LPS [11,12]. By adding 1%, 2%, and 3% PxB to the PSUR/LPS mixture, we noticed that concentrations of 1% and 2% did not prevent surfactant inactivation by LPS. Only a 3% concentration of PxB effectively counterbalanced the inhibitory effect of LPS at 5%, 10%, and 20%, allowing *γmin* to be reduced to values typical of the functional surfactant (<5 mN/m).

It was suggested that PxB leads to the acyl chain fluidisation of LPS. This ability was used to develop synthetic anti-LPS peptides, which are designed to strongly bind to the lipid A part of LPS [50]. Neutralisation of LPS by these peptides, structurally related to PxB, is associated with a fluidisation of the LPS acyl chains and a dramatic change in the LPS aggregate type from cubic into multilamellar, with an increase in the aggregate size. Other groups of synthetic amphiphilic polymers with endotoxin-neutralising properties also bind and cause a pseudoaggregate formation, resulting in LPS neutralisation/detoxification [51].

When added to the PSUR/LPS mixture, PxB also bound to the PSUR itself. The addition of 2% PxB significantly improved the surface properties of surfactant and increased its resistance to inactivation with albumin [11]. The favourable *in vitro* effect of PxB is explained by its ability to bridge PL membranes, making it easier to develop more-resistant multilayer structures [10]. PxB also increases the resistance of the surfactant to meconium, and combined treatment with PSUR/PxB is effective against the systemic spread of *E. coli* [12,13].

The beneficial effect of PxB is attributed to its ability to connect lipid vesicles and enlarge the surface-associated surfactant reservoir in the hypophase [20], making PLs available for surface adsorption at the “bubble” surface. Moreover, PxB promotes the intermembrane transfer of PLs [52].

Our experiments revealed significant structural changes caused by PxB addition to the PSUR/LPS mixture. The connection of two methods, polarised light microscopy and SAXS, revealed a swelling lamellar phase of the PSUR reaching spacings of up to ~13 nm as a result of the intercalation of LPS (applied at a concentration of 10%) to the lipid bilayer. Our experiments did not quantify the fraction of LPS that penetrated into multilamellar vesicles but confirmed a significant dependence on the incubation time. Cationic PxB appeared as a powerful inhibitor of the structural disorder induced by LPS. Irrespective of the incubation time, 2% PxB was a satisfactorily high concentration to recover multilamellar packing with a spacing of *d* ~ 7–8 nm, close to that observed in the pure PSUR dispersion (*d* ~ 8.4 nm). PxB at a higher dose, that is, a 3% concentration, caused a significant reduction to *d* ~ 6.8 nm. Our experiments did not allow us to distinguish between the effects of PxB on the thicknesses of the lipid bilayer and water layer. The amphipathic character of the molecule predestines its insertion into the lipid bilayer, eventually causing its thinning, as proposed in the Shai–Matsuzaki–Huang model for the antimicrobial mechanism of peptides [53,54,55]. We also cannot exclude pore formation in the bilayer, although both experimental methods showed that lamellar packing was preserved. Our structural results and analysis of surface charges in the PSUR/LPS membrane indicated that positively charged hydrophilic residues of PxB compensated their charges by interaction with negatively charged groups of both LPS and LPs (discussed above), which reduced the electrostatic repulsion between bilayers and resulted in a reduction in the water layer thickness and consecutively in the repeat distance of the lamellar packing. The information on PxB behaviour documented in the literature together with results reported above demonstrate that the mechanism explaining the antimicrobial activity in our systems is not disintegration of the membrane due to pore formation. On the contrary, our findings are in favour of a mechanism of membrane condensation forming multilamellar lipid stacks, as shown recently in a model of a bacterial membrane and antimicrobial peptide of a cecropin A–melittin hybrid [56]. 

We found that, while a 2% concentration of PxB was a sufficient dose to reverse reliably structural changes induced by 10% LPS, it was not sufficiently high for full functional recovery of the surfactant.

In summary, LPS prevents surfactants at low concentrations from reaching low surface tension during area compression, mimicking exhalation. LPS, via binding to the lipid bilayer of the PSUR, disturbs its lamellar structure by swelling, allowing additional water to penetrate into interlamellar space. The observed structural changes were attributed to the surface charge unbalance of surfactant lipid bilayers due to LPS insertion. The cationic molecules of PxB acted as an inhibitor of structural changes induced by LPS, and as a result of charge compensation, the original lamellar packing was restored. In addition, our experiments indicate the great importance of setting the right dose of PxB when healing bacterial infections in order to inhibit the effect of LPS and to avoid any destruction of the membrane.

## 4. Materials and Methods

### 4.1. Chemicals

Curosurf (poractant alpha; Chiesi Pharmaceutici, Parma, Italy) is a PSUR provided in the concentration of PLs: 80 mg/mL. It was suspended in 0.9% NaCl at a PL concentration of either 1.75 or 10 mg/mL (for the structural study). LPS (*E. coli*, O55:B5; Santa Cruz Biotechnology, Inc., Dallas, TX, USA) consisting of a lipid A moiety linked to an antigenic *O*-polysaccharide, was dissolved in 0.9% NaCl to a stock solution of 1 or 2.5 mg/mL and was kept frozen. The stock solution of PxB (polymyxin B sulphate; AppliChem GmbH, Darmstadt, Germany) was prepared in sterile distilled water at concentrations of 5 and 50 mg/mL.

#### Mixtures

The PSUR/LPS and PSUR/LPS/PxB mixtures were prepared by adding LPS to the PSUR at concentrations of 1%, 5%, 10%, and 20% (by mass of surfactant PLs, *w*/*w*) and by adding PxB at concentrations of 1%, 2%, and 3% (*w*/*w*) to the porcine surfactant. Each sample was gently mixed and incubated at 37 °C for either 30 min or 2 h.

### 4.2. Evaluation of Surface Activity

Surface activity was evaluated using a pulsating bubble surfactometer (General Transco Inc., Seminole, FL, USA). Approximately 40 µL of test fluid was filled in an acrylic sample chamber. A bubble of minimum radius (r = 0.4 mm) was created at 37 °C, and it was maintained at a minimal size for 30 s. Next, pulsation was started at a cycling rate of 20 rpm. The bubble radius varied between 0.4 and 0.55 mm (corresponding to an area compression of 50%). Pressure across the surface of the bubble was continuously recorded with a microprocessor, and values for surface tension at the minimum and maximum bubble sizes were calculated using the Laplace equation [46]. Data was digitally recorded. Measurements were made five times for each sample.

### 4.3. SAXS and WAXS Experiments

For X-ray measurements, the samples were centrifuged for 20 min at 13,400 rpm (~12,100× *g*). Sediment of the samples was carefully transferred into special glass capillaries (WJM-Glas/Muller GmbH, Berlin, Germany) with diameters of 1.5 mm for X-raying. X-ray experiments were performed at BL11-NCD beamline at Alba Synchrotron, Barcelona, Spain using monochromatic radiation with a wavelength of *λ* = 0.12 nm. The sample in the vertically placed capillary was equilibrated at a temperature of 37 °C before being exposed to radiation. SAXS and WAXS images were collected simultaneously. The patterns were recorded using the SAXS pixel detector ImXPAD-S70 and the WAXS LX-255 HS detector. The raw data were normalised against the incident beam intensity. The *q* range of the SAXS patterns was calibrated using silver behenate, and chromium trioxide was used for WAXS data calibration. The patterns were corrected for the used solvent. Each peak of the SAXS region was fitted with a Lorentzian curve above a linear background. The Lorentzian is defined by *I* = *I_n_*/(1 + ((*q* − *q_n_*)/*Δq_n_*)^2^), where *q_n_* (*n* = 1, 2…) are positions of maxima, *I_n_* is the intensity of the peak, and *∆q_n_* is its half width at half maximum. The repeat distance *d* was determined from *d* = 2·*π*/*k*, where *k* is the slope of *q*(nm^−1^) = *f*(*n*) including the origin (0,0). The uncertainty in *d* is expressed from the standard deviation of the slope.

### 4.4. Optical and Polarised Light Microscopy

Optical and polarised light microscopic studies were performed with a polarised light Nikon Eclipse LV100N POL microscope at 20 °C. Photographic images (micrographs) were recorded with a CCD Nikon DS-Fi2 camera.

### 4.5. Statistics

Data are expressed as means ± SEM. STATISTICA Cz 10 was used for data analysis. Between-group differences were examined by ANOVA followed by a Newman–Keuls post hoc test. A *p*-value of <0.05 was considered to be statistically significant.

## Supplementary Materials

Supplementary materials can be found at http://www.mdpi.com/1422-0067/19/7/1964/s1.

## Abbreviations

LPS	Lipopolysaccharide
PxB	Polymyxin B
PSUR	Modified porcine pulmonary surfactant
SAXS	Small-angle X-ray scattering
WAXS	Wide-angle X-ray scattering
*γmin*	Minimum surface tension
*γmax*	Maximum surface tension
PL	Phospholipid
