# Supplementary material for: The Perturbation of Pulmonary Surfactant by Bacterial Lipopolysaccharide and Its Reversal by Polymyxin B: Function and Structure
Source: Int J Mol Sci. 2018 Jul 5;19(7):1964. doi: 10.3390/ijms19071964 (PMC6073772; doi:10.3390/ijms19071964)
Supplement: Supplementary file 1 [file ijms-19-01964-s001.pdf]

Supplementary material

The perturbation of pulmonary surfactant by bacterial lipopolysaccharide and its reversal by polymyxin B: function and structure

Kolomaznik Maros[a,b], Liskayova Gilda[c], Kanjakova Nina[c], Hubcik Lukas[c], Uhrikova Daniela*[c], Calkovska Andrea[a,b]

[a]Martin Biomedical Center and [b]Department of Physiology, Jessenius Faculty of Medicine in Martin; Comenius University in Bratislava, Slovakia; [c]Department of Physical Chemistry of Drugs, Faculty of Pharmacy, Comenius University in Bratislava, Slovakia

postal address of affiliation

[a,b]Department of Physiology and BioMed, Jessenius Faculty of Medicine, Comenius University, Mala Hora 4C, SK-036 01 Martin, Slovakia

[c]Department of Physical Chemistry of Drugs, Faculty of Pharmacy, Comenius University, Odbojarov 10, SK-832 32 Bratislava 3, Slovakia

e-mail addresses

Kolomaznik Maros - maros.kolomaznik@uniba.sk
Liskayova Gilda - liskayova@fpharm.uniba.sk
Kanjakova Nina– kanjakova2@uniba.sk
Hubcik Lukas - hubcik@fpharm.uniba.sk
Uhrikova Daniela - daniela.uhrikova@fpharm.uniba.sk
Calkovska Andrea - andrea.calkovska@jfmed.uniba.sk

Corresponding author

*Daniela Uhrikova, prof.
Department of Physical Chemistry of Drugs
Faculty of Pharmacy
Comenius University
Odbojarov 10
SK-832 32 Bratislava 3
Slovakia
daniela.uhrikova@fpharm.uniba.sk

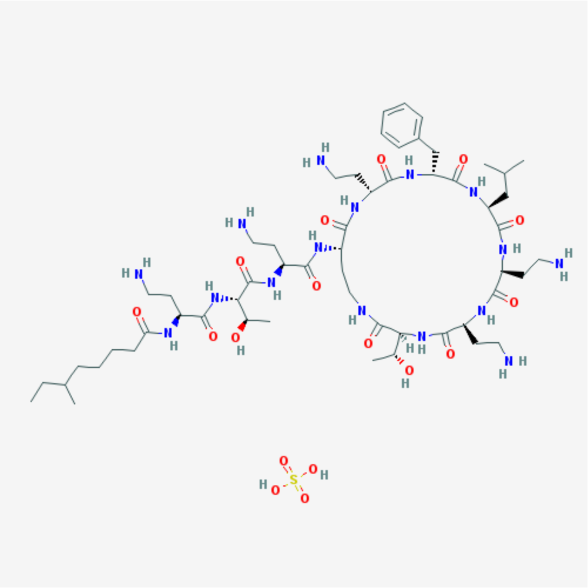

Figure S1. Structure of polymyxin B (PxB).

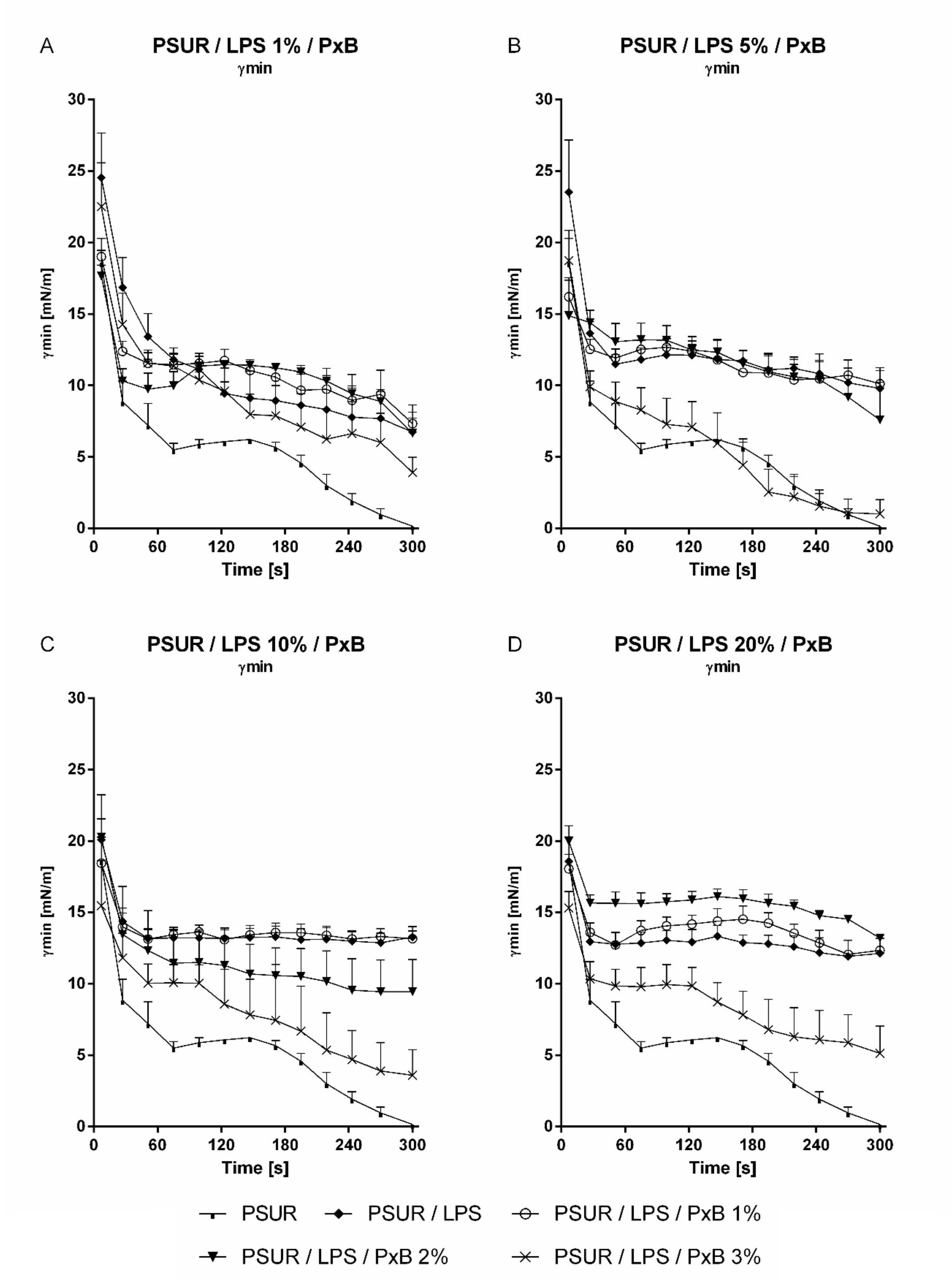

Figure S2. Minimum surface tension (*γmin*) of modified porcine surfactant (PSUR) at phospholipid concentration 1.75 mg/ml with lipopolysaccharide (LPS) 1 % and polymyxin B (PxB) 1, 2, 3 % (A), LPS 5 % and PxB 1, 2, 3 % (B), LPS 10 % and PxB 1, 2, 3 % (C) and LPS 20 % and PxB 1, 2, 3 % (D) during the whole period of pulsation 5 minutes. Values are given as mean±SEM.

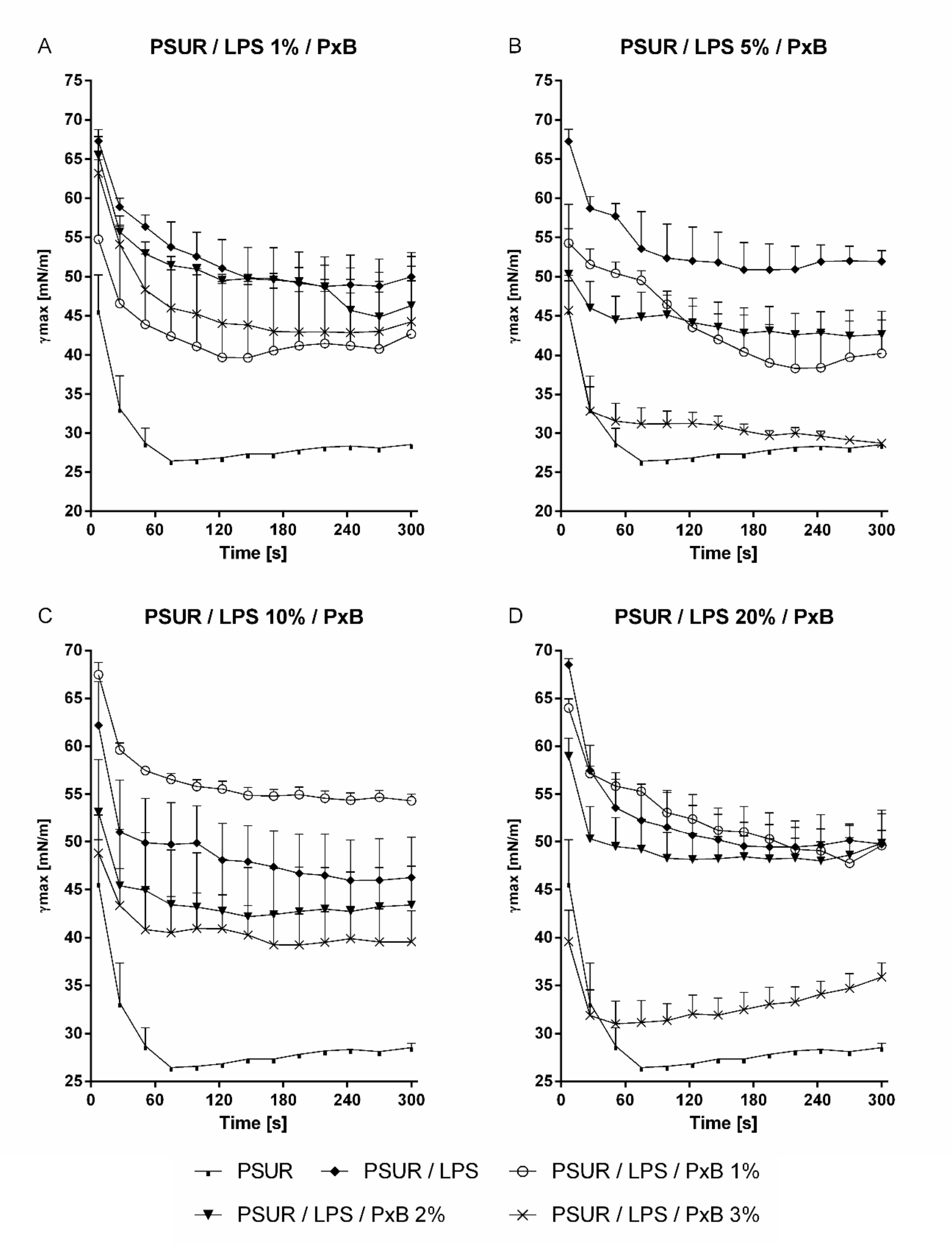

Figure S3. Maximum surface tension (*γmax*) of modified porcine surfactant (PSUR) at phospholipid concentration 1.75 mg/ml with lipopolysaccharide (LPS) 1 % and polymyxin B (PxB) 1, 2, 3 % (A), LPS 5 % and PxB 1, 2, 3 % (B), LPS 10 % and PxB 1, 2, 3 % (C) and LPS 20 % and PxB 1, 2, 3 % (D) during the whole period of pulsation 5 minutes. Values are given as mean±SEM.

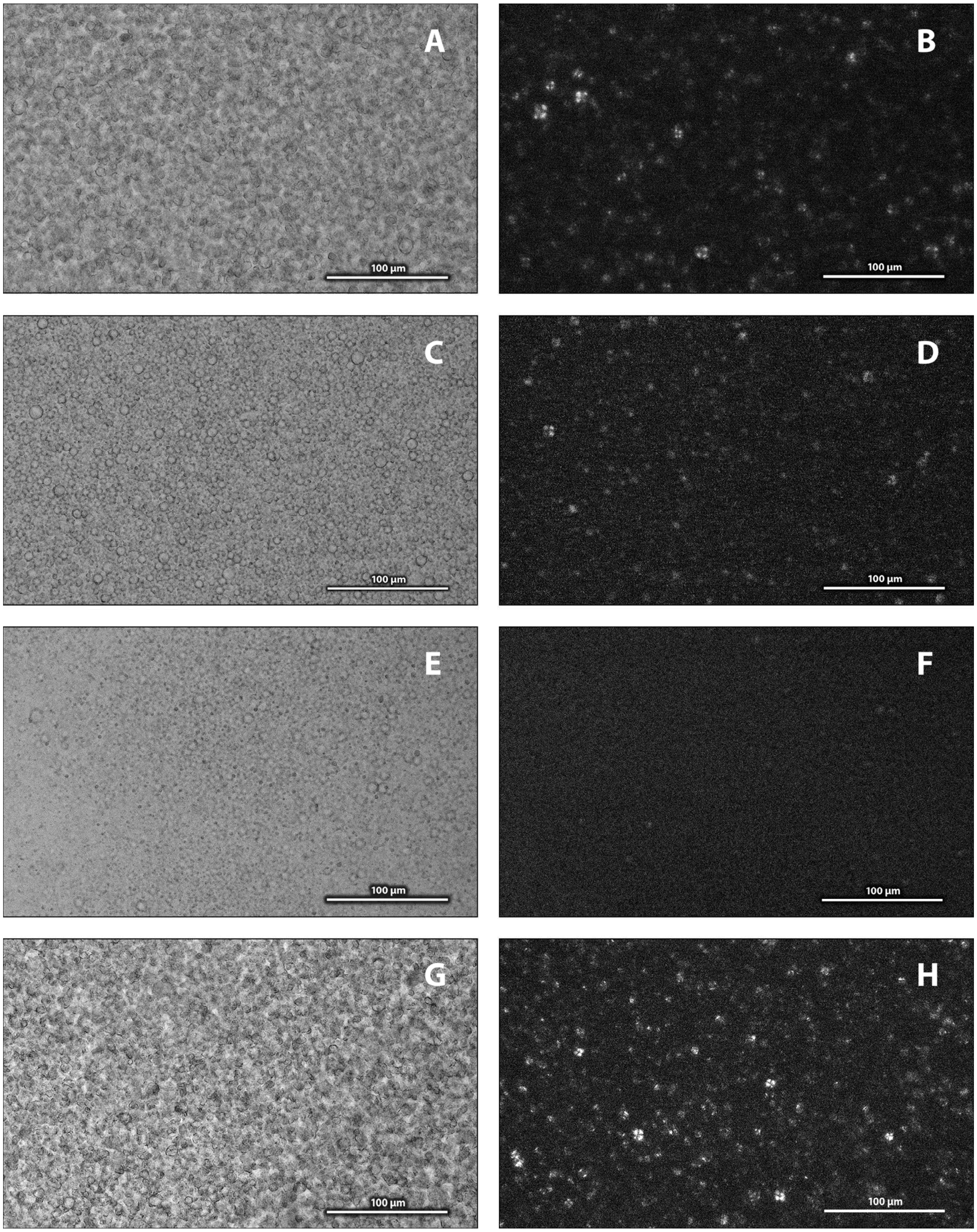

Figure S4. Microscopy photos recorded in normal and polarized light for aqueous dispersion of modified porcine surfactant (PSUR) (A and B); PSUR and 10 % LPS incubated for 30 min at 37 ^{o}C (C and D); PSUR and 10 % LPS incubated for 2 hours at 37 ^{o}C (E and F); PSUR/ 10 % LPS and 3 % PxB (G and H).

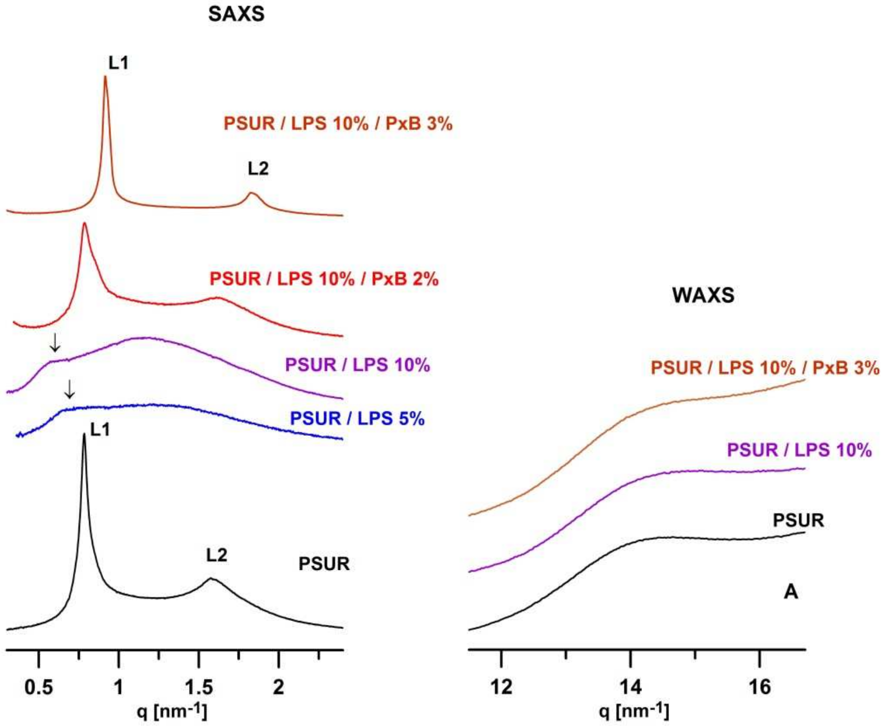

Figure S5. SAXS patterns of modified porcine surfactant (PSUR); PSUR and 5 % LPS; PSUR and 10 % LPS incubated for 2 hours; PSUR/10 % LPS and 2 % PxB; PSUR/10 % LPS and 3 % PxB at 37 ^{o}C. An arrow indicates the first order peak of a lamellar phase with *d* ~ 12-13 nm. WAXS patterns of PSUR, PSUR/10% LPS, PSUR/10 % LPS and 3 % PxB (37°C).

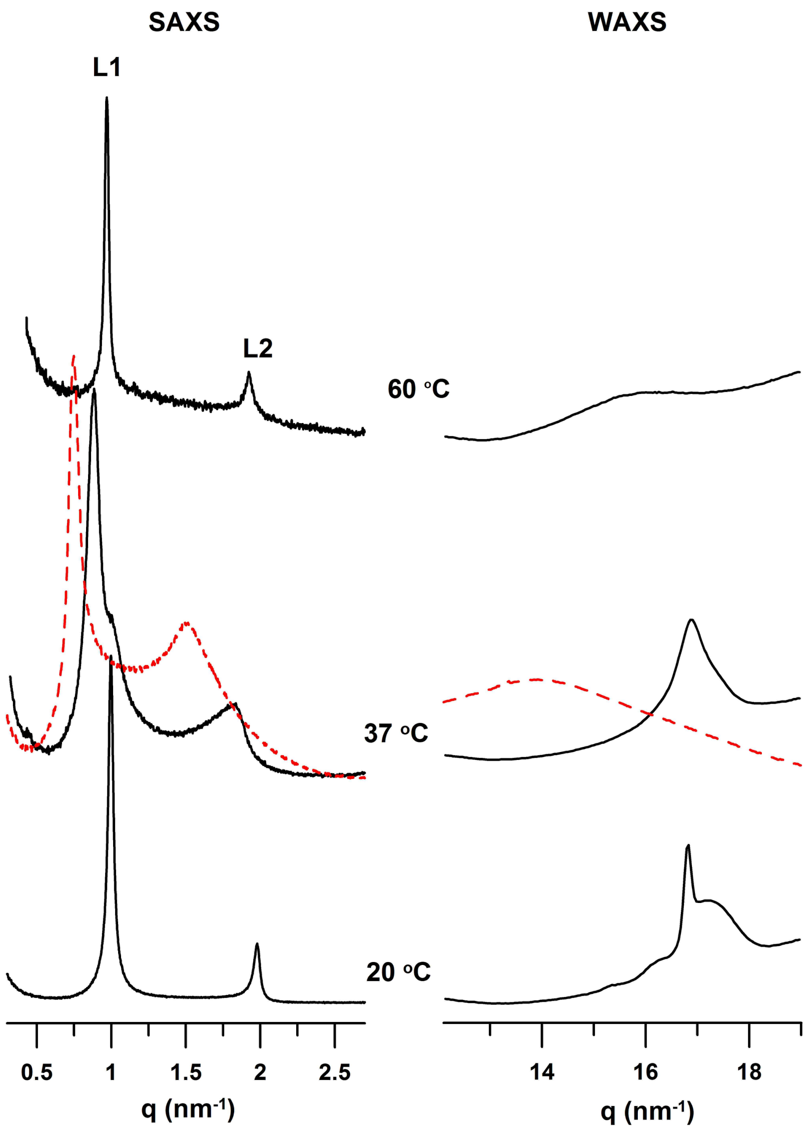

Figure S6. SAXS/WAXS patterns of fully hydrated DPPC: tilted gel $L_{\beta'}$ phase at 20 °C (repeat distance *d* ~ 6.3 nm); rippled gel P_{β} phase at 37 °C (repeat distance *d* ~ 7.3 nm); liquid-crystalline L_{α} phase at 60 °C (repeat distance *d* ~ 6.6 nm). Dashed red lines represent patterns of PSUR at 37 °C.
